# Potential Biomarkers for Predicting the Risk of Developing Into Long COVID After COVID‐19 Infection

**DOI:** 10.1002/iid3.70137

**Published:** 2025-01-24

**Authors:** Zhiyong Hou, Yu Ming, Jun Liu, Zhong Wang

**Affiliations:** ^1^ Institute of Basic Research in Clinical Medicine China Academy of Chinese Medical Sciences Beijing China

**Keywords:** COVID‐19, immune cell infiltration, long COVID, machine learning algorithms, modular pharmacology platform

## Abstract

**Background:**

Long COVID, a heterogeneous condition characterized by a range of physical and neuropsychiatric presentations, can be presented with a proportion of COVID‐19‐infected individuals.

**Methods:**

Transcriptomic data sets of those within gene expression profiles of COVID‐19, long COVID, and healthy controls were retrieved from the GEO database. Differentially expressed genes (DEGs) falling under COVID‐19 and long COVID were identified with R packages, and contemporaneously conducted module detection was performed with the Modular Pharmacology Platform (http://112.86.129.72:48081/). The integration of both DEGs and differentially expressed module‐genes (DEMGs) regarding long COVID and COVID‐19 was intersected by following Gene Ontology (GO), Kyoto Encyclopedia of Genes and Genomes (KEGG), and Gene Set Enrichment Analysis (GSEA).

**Results:**

There were 11 and 62 differentially expressed modules, 1837 and 179 DEGs, as well as 103 and 508 DEMGs acquiring identified for both COVID‐19 and long COVID, notably enriched in the immune‐correlated signaling pathways. The immune infiltrating cells of long COVID and COVID‐19 were comparatively and respectively assessed via CIBERSORT, ssGSEA, and xCell algorithms. Subsequently, the screening of hub genes involved employing the SVM‐RFE, RF, XGBoost algorithms, and logistic regression analysis. Among the 67 candidate genes were processed with machine learning algorithms and logistic regression, a subgroup consisting of CEP55, CDCA2, MELK, and DEPDC1B, was at last identified as potential biomarkers for predicting the risk of the progression into long COVID after COVID‐19 infections. The predicting performance of the potential biomarkers was quantified with a ROC value of 0.8762542, which proved the combination of potential biomarkers provided the highest performance.

**Conclusions:**

In summary, we identified a subgroup of potential biomarkers for predicting the risk of the progression into long COVID after COVID‐19 infection, which could be partly elucidation of the associated molecular mechanisms for long COVID.

## 1 Introduction

Reports from the World Health Organization (WHO) indicate that around 25% of individuals with COVID‐19 continue to experience symptoms 4–5 weeks after diagnosis, and approximately 1 in 10 have continuing symptoms after 12 weeks, which is known as long COVID [1]. Individuals with long COVID have reported a range of symptoms, including, but not limited to, fatigue, anosmia (loss of the sense of smell), memory loss, gastrointestinal distress, and shortness of breath. These symptoms significantly impact individuals' routine work and daily activities [2, 3, 4]. In a large cohort study [5], findings revealed that 76% of recovered patients, 6 months post‐acute infection, displayed at least one persistent symptom among 1733 patients discharged from the hospital due to COVID‐19. Another study involving 3762 patients from 56 countries, with a nearly 7‐month follow‐up, revealed that over 91% of respondents experienced a recovery time exceeding 35 weeks. Fatigue and postexertional cognitive dysfunction were identified as the most prevalent symptoms after 6 months [6].

Although the era of global pandemic outbreaks of COVID‐19 has temporarily subsided, the novel variant will persist for an extended period, establishing the imperative that post‐COVID‐19 health issues necessitate continuous and chronic management for an extended duration [7]. Long COVID constitutes a series of persistent symptoms following recovery from SARS‐CoV‐2 infection, involving multiple organ systems throughout the body and bearing long‐term consequences for the patient's physical and mental health [8]. The persistent presence of SARS‐CoV‐2 may be a significant driver of the pathological mechanisms underlying long COVID. Weeks to months after acute SARS‐CoV‐2 infection, viral RNA and proteins have been detected in various tissues [9, 10, 11, 12, 13]. These viral components may interact with host pattern recognition receptors, triggering cytokine release and initiating inflammatory responses. The interactions potentially mediate host metabolic, genetic, and epigenetic factors, triggering the dysregulation of signaling pathways [14], which in turn results in widespread immune responses, primarily marked by inflammation and immune dysregulation. Current research on biomarkers for long COVID have focused on complement and coagulation‐related proteins [15, 16], including COMP, PLG, TPM2, TPM1, APOA4, and LRP1. Other studies have examined immune‐related proteins or metabolites, such as IFN‐β, IFN‐λ1, IL‐1α, IL‐6, TNF‐α, and IP‐10 [3, 17].

Currently, biomarkers for long COVID and prognostic risk models are inadequately explored, and the molecular mechanisms underlying the progression from COVID‐19 to long COVID remain unclear. There is a critical need to investigate biomarkers that facilitate the diagnosis of the transition from COVID‐19 to long COVID. Our study identified CEP55, CDCA2, MELK, and DEPDC1B as potential biomarkers for the progression from COVID‐19 to long COVID, with the mechanisms of this transition intimately associated with immune and virus. These biomarkers serve as crucial targets, providing fresh insights into prevention, diagnosis, and treatment.

## 2 Methods

### 2.1 Raw Data Acquisition and Preprocessing

Microarray data sets, which originated from blood samples, including GSE157103 on platform GPL24676 (COVID‐19 = 100, healthy controls = 26, male = 76, female = 40, median age = 61.8), GSE224615 on platform GPL20301 (long COVID = 23, healthy controls = 13, male = 16, female = 20, median age = 58, collection point time = 8 months), and GSE169687 on platform GPL24676 (long COVID = 134, healthy controls = 14, male = 43, female = 40, median age = 46, collection point time = 12, 16, 24 weeks), were obtained from the Gene Expression Omnibus (GEO) database (https://www.ncbi.nlm.nih.gov/geo/). Subsequently, the raw gene expression profiles were normalized using R software (version 4.3.2).

### 2.2 Differential Expression Genes (DEGs) Analysis

Based on setting the threshold values to | logFC | > 0.5 and *p* < 0.05, the “limma” package was utilized to analyze COVID‐19 samples, long COVID samples, and control samples from the GSE157103 and GSE224615 data sets. Statistically significant DEGs of COVID‐19 and long COVID were eventually obtained.

### 2.3 Modular Pharmacological Computational Platform

Gene expression matrices originated from the GSE157103 and GSE224615 data sets were analyzed within the Modular Pharmacology Platform (http://112.86.129.72:48081/). On this platform, the gene co‐expression network construction and module detection were implemented via the WGCNA algorithm [18], and the topological overlap measure and Dynamic Hybrid Tree Cut algorithm were applied for average linkage hierarchical clustering, partitioning the branches of dendrogram as module, followed by employing module discrimination analysis [19]. Zsummary value, MT value, and ME value were to assess whether the module was conserved or preserved; specifically, in this research, modules with a *Z*_summary_ value [20, 21, 22] ≤ 2 were considered differential modules. Whereafter the DEMGs were selected accordingly.

(1) $$Z_{\text{summary}}=\frac{\mathrm{median}(Z_{\text{meanCor},Z\text{meanAdj}},Z_{\text{poropvarExpl}},Z_{\text{meanKME}})+\mathrm{median}({Z\text{cor}.}_{\text{KM}},{Z\text{cor}.}_{\text{KME}},{Z\text{cor}.}_{\text{cor}})}{2}$$

(2) $$e(G)=\sum_{v\epsilon V}e(V)$$

(3) $$\mathrm{MT}=\frac{3}{4}Z_{\mathrm{summary}}-\frac{1}{4}e(G)$$

(4) $$\mathrm{ME}=\frac{3}{4}Z_{\mathrm{summary}}+\frac{1}{4}e(G)$$

### 2.4 Enrichment Analysis

To investigate the biological functional categories and the potential mechanisms of the combination of DEGs and CEMGs, we employed the “clusterProfiler” package [23], the “org.Hs.eg.db” package, and the “c5.all.v7.0.entrez.gmt” file to conduct Gene Ontology (GO) function enrichment analysis, Kyoto Encyclopedia of Genes and Genomes (KEGG) pathway analysis, and Gene Set Enrichment Analysis (GSEA). The GO terms were categorized into three main classes: biological process (BP), cellular component (CC), and molecular function (MF). Statistically significant cut‐off thresholds were set at adjusted *p* < 0.05 and *q* < 0.05.

### 2.5 Evaluating of Immune Infiltrating Cells

The CIBERSORT algorithm, taking “CIBERSORT.R” and the LM22 signature matrix as the foundation, was to assess the relative abundance of 22 types of immune cells in each sample [24]. Simultaneously, the “GSVA” package, the “xCell” package, the “cellMarker.csv” file, and the “immune.gmt” file were employed to uncover the expression levels of diverse immune cells and immune functions [25, 26].

### 2.6 Machine Learning Algorithms

Candidate genes were initially obtained by intersecting and integrating DEGs and DEMGs regarding long COVID and COVID‐19 via the “venn” and “VennDiagram” packages. Subsequently, we employed three types of machine learning algorithms: Random Forest (RF) [27], Support Vector Machine‐Feature Recursive Elimination (SVM‐RFE) [28], and eXtreme gradient boosting (XGboost) [29] algorithms, which were to further investigate the expression data of candidate genes in COVID‐19 and long COVID, by means of the “glmnet” package, “randomForest” package, “e1071” package, and “xgboost” packages. The MeanDecreaseGini value greater than 0.2 was set as the statistic threshold in the RF algorithm.

### 2.7 The Prediction Model for the Potential Biomarkers of Long COVID

The outcomes for candidate genes applied to the machine learning algorithms were visualized by the “ggsankey” package. Moreover, the leveraging of the logistic regression approach for being analytically positioned as further screening of evaluating the independent predictive value of genes, after which the hub genes were acquired. Simultaneously, the expression of the machine learning algorithms result‐gene, for both COVID‐19 and long COVID, which originated from the disease and control samples, was assessed using the “pheatmap” package, followed by gene‐to‐gene correlation analysis. To validate the predictive performance of hub genes associated with the development of long COVID, the “rms” package was utilized to construct a nomogram model to assess the risk for long COVID. Calibration curves, based on the “Hmisc” package, and ROC curves, based on the “ROCR” package, were employed to evaluate the predictive power of the nomogram model.

## 3 Results

### 3.1 Screening of DEGs and DEMGs

**Figure 1:**
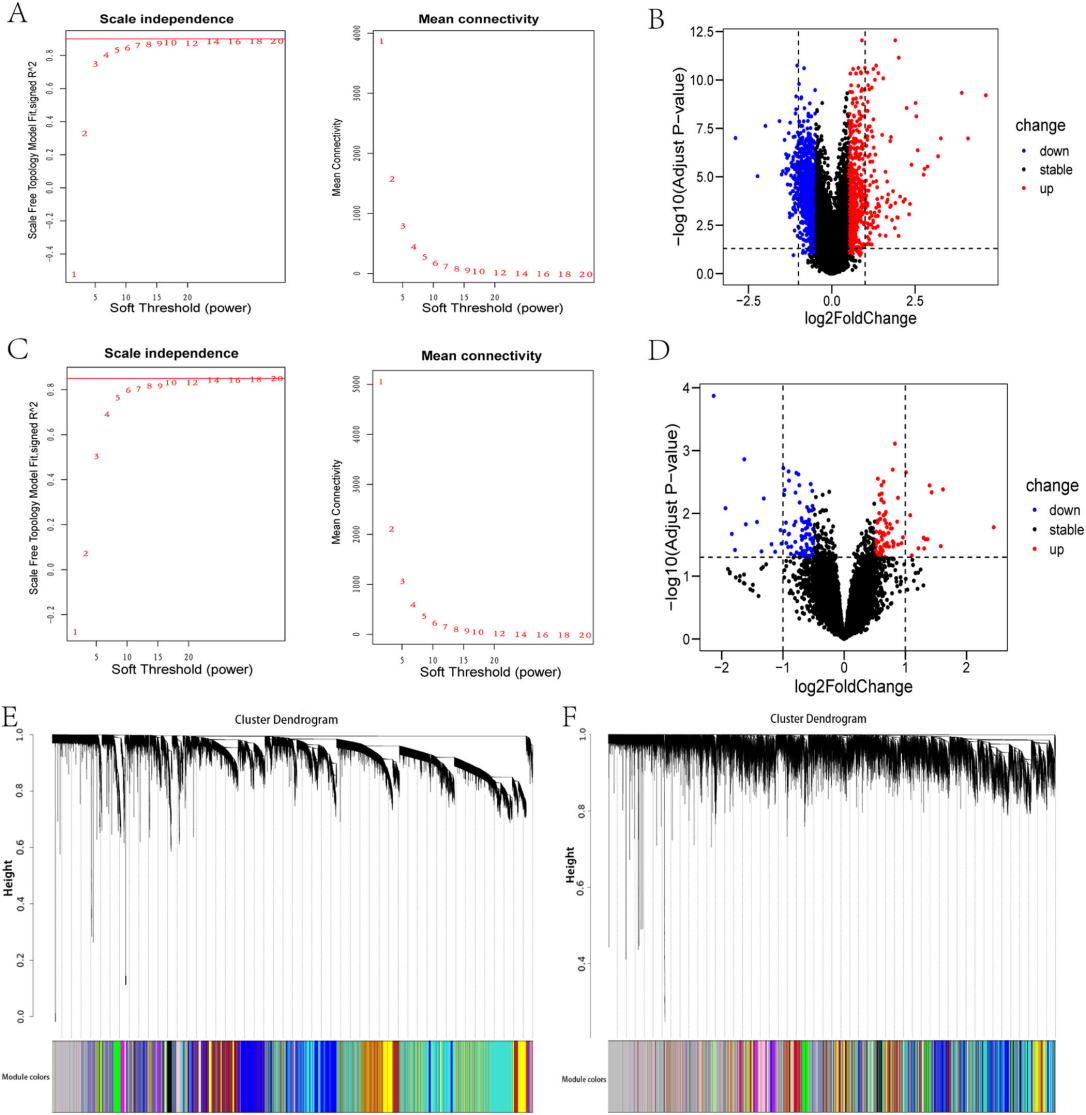
Identification of DEGs and CDMGs, as well as Soft Threshold (power) selection. Scale Independence and mean connectivity of COVID‐19 (A) and long COVID (C); the volcano plot of COVID‐19 (B) and long COVID (D); (B) clustering dendrogram of COVID‐19 (E) and long COVID (F).

The results of the differential expression analysis of the GSE157103 data set unveiled a total of 1837 DEGs (Figure 1B), and of the GSE224615 data set, 179 DEGs (Figure 1D). Following the import of genes from GSE157103 and GSE224615 expression data into the Modular Pharmacology Platform, soft thresholds of 9 and 18 were selected, which aimed to construct a scale‐free network that adheres more effectively to the scale‐free architecture (Figure 1A,C). After identifying co‐expression modules, modules were detected based on the clustering dendrogram (Figure 1E,F), and relationships between modules were illustrated via heatmap (Supporting Information S1). A total of 11 and 62 modules were detected as COVID‐19 and long COVID differential modules, comprising 103 and 509 DEMGs on the Modular Pharmacology Platform (Supporting Information [Link], [Link], [Link], [Link], [Link], [Link]).

### 3.2 Candidate Genes Acquisition and Immune Infiltrating Cells Analysis

**Figure 2:**
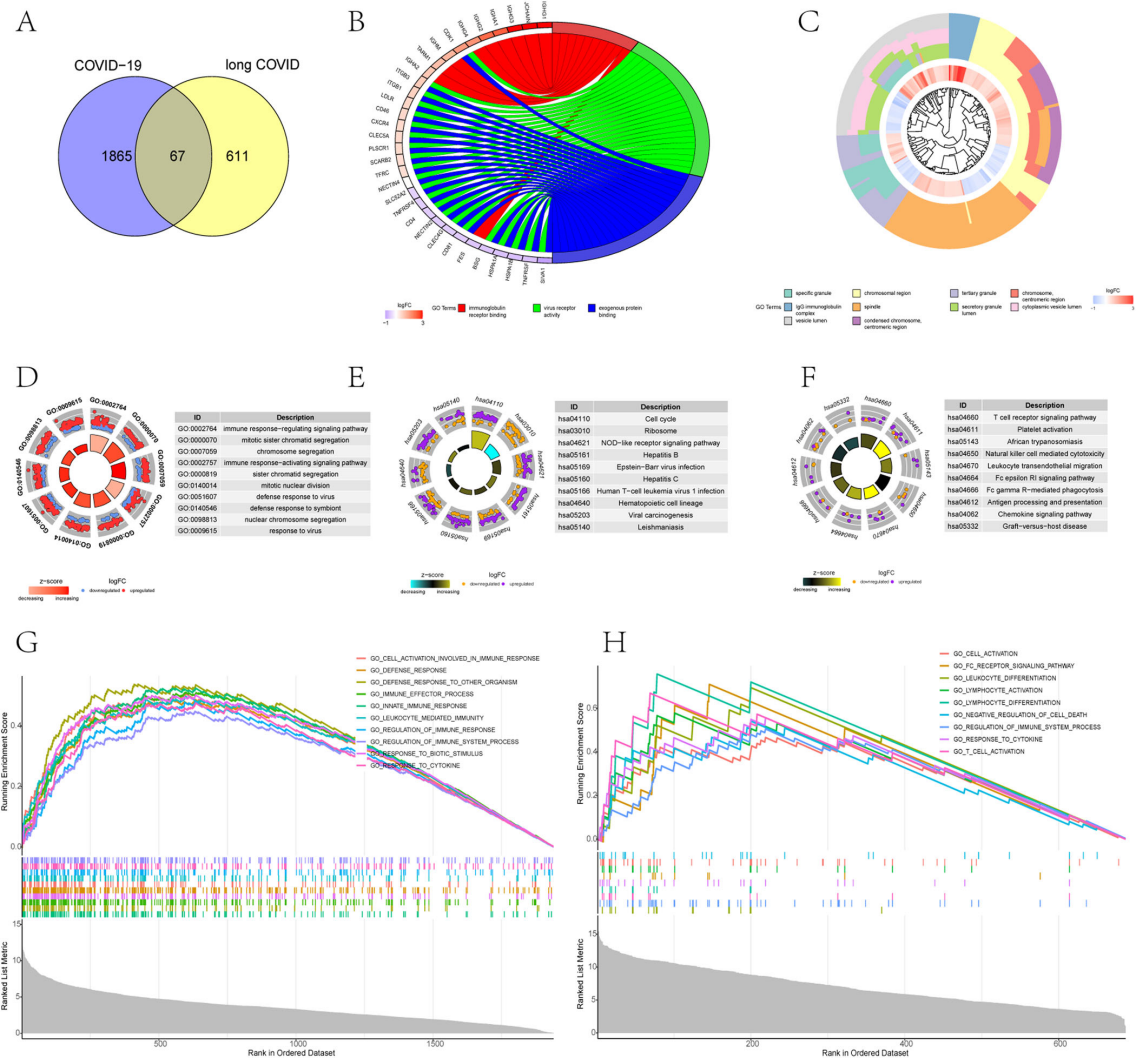
GO, KEGG, and GSEA enrichment analysis. (A) Venn diagram of COVID‐19 and long COVID. (B−D) Gene ontology (GO) terms in molecular function (MF), cellular component (CC), and biological process (BP) for COVID‐19. Kyoto Encyclopedia of Genes and Genomes (KEGG) pathways for COVID‐19 (E) and long COVID (F). Gene Set Enrichment Analysis (GSEA) for COVID‐19 (G) and long COVID (H).

By overlapping of DEGs and DEMGs, 67 candidate genes were identified as the result (Figure 2A; Supporting Information S8). Enrichment analysis of both COVID‐19 and long COVID indicated a remarkable correlation with immunity (Supporting Information [Link], [Link], [Link], [Link], [Link]). In terms of BP, the enriched terms included immune response‐activating signaling pathways, immune response‐regulating signaling pathways, defense response to virus, response to virus, innate immune response in mucosa, regulation of response to cytokine stimulus, among others (Figure 2D). Furthermore, analyses of CC and MF revealed immune‐related terms such as IgG immunoglobulin complex, primary lysosome, immunoglobulin complex, circulating, IgA immunoglobulin complex, immunoglobulin receptor binding, virus receptor activity, and others (Figure 2B,C). KEGG pathway analysis of COVID‐19 and long COVID demonstrated significant enrichment in pathways, for example, NOD‐like receptor signaling, neutrophil extracellular trap formation, coronavirus disease [COVID‐19], Epstein−Barr virus infection, Fc epsilon RI signaling pathway, Fc gamma R‐mediated phagocytosis, and cAMP signaling pathway (Figure 2E,F). GSEA results between COVID‐19 and long COVID indicated associations between the GO_INNATE_IMMUNE_RESPONSE, GO_DEFENSE_RESPONSE_TO_OTHER_ORGANISM, GO_IMMUNE_EFFECTOR_PROCESS, GO_DEFENSE_RESPONSE, and GO_REGULATION_OF_IMMUNE_SYSTEM_PROCESS pathways with immunity (Figure 2G,H).

**Figure 3:**
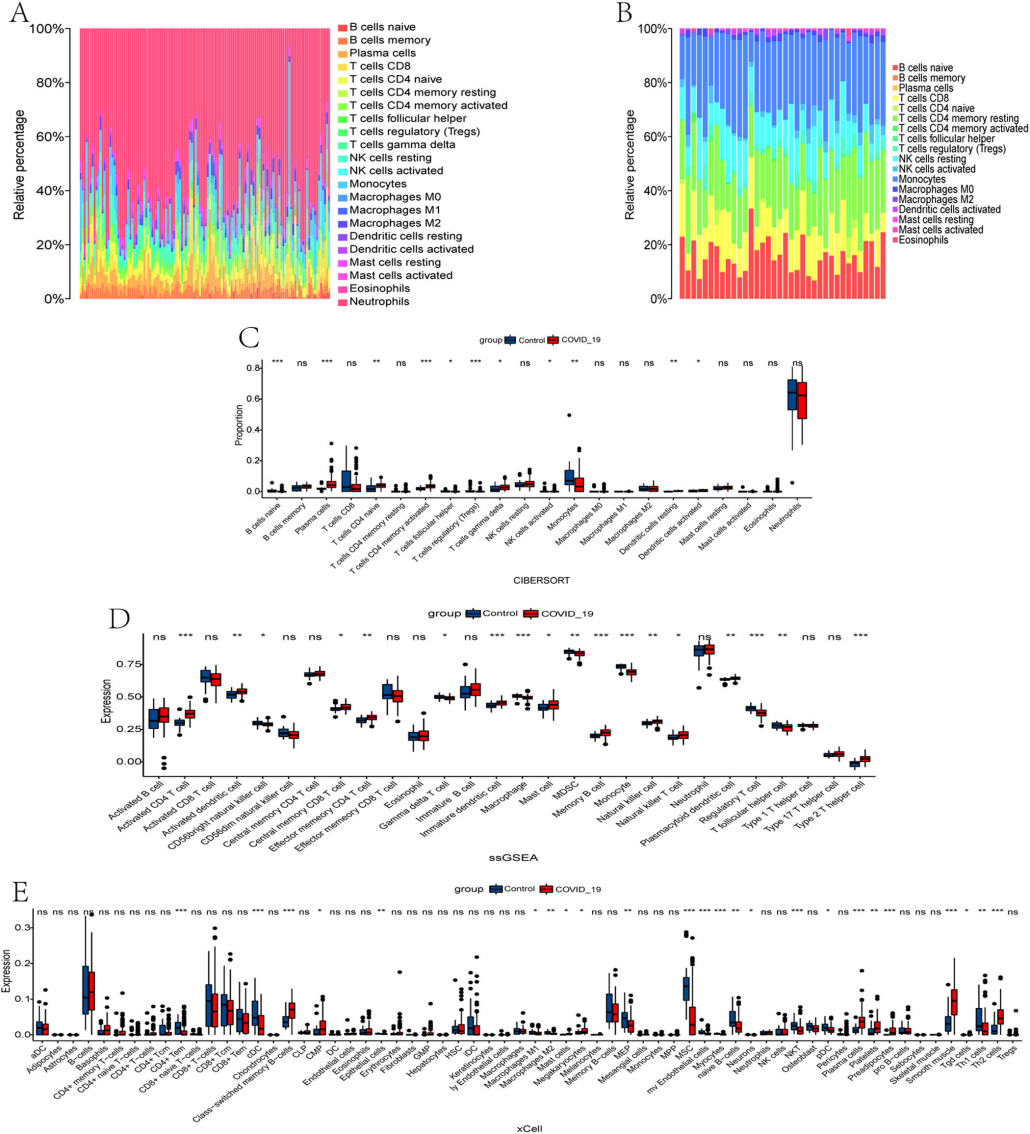
Comparison of immune infiltration between both COVID‐19 and long COVID groups and control groups. The proportion of 22 kinds of immune cells in all samples of COVID‐19 (A) and long COVID (B). (C−E) Proportion and expression of immune cells via CIBERSORT, ssGSEA, and xCell analysis for COVID‐19 (ns, no significance, *p < 0.05, **p < 0.01, ***p < 0.001).

To further explore the relationship between both COVID‐19 and long COVID, as well as immune cell infiltration and function, GSVA, CIBERSORT, xCell, and ssGSEA were applied to estimate expression differences. CIBERSORT was employed to ascertain the enriched proportion of 22 distinct immune cell types in all the samples (Figure 3A,B). Immune infiltrating cells analysis revealed alterations in immune cell expression between COVID‐19 and control samples, with various cell subtypes, including macrophages, T cell CD4 memory activated, activated CD4 T cells, memory B cells, and so forth, exhibiting higher expression levels in COVID‐19 samples (Figure 3C−E).

### 3.3 Machine Learning Algorithms for Screening

**Figure 4:**
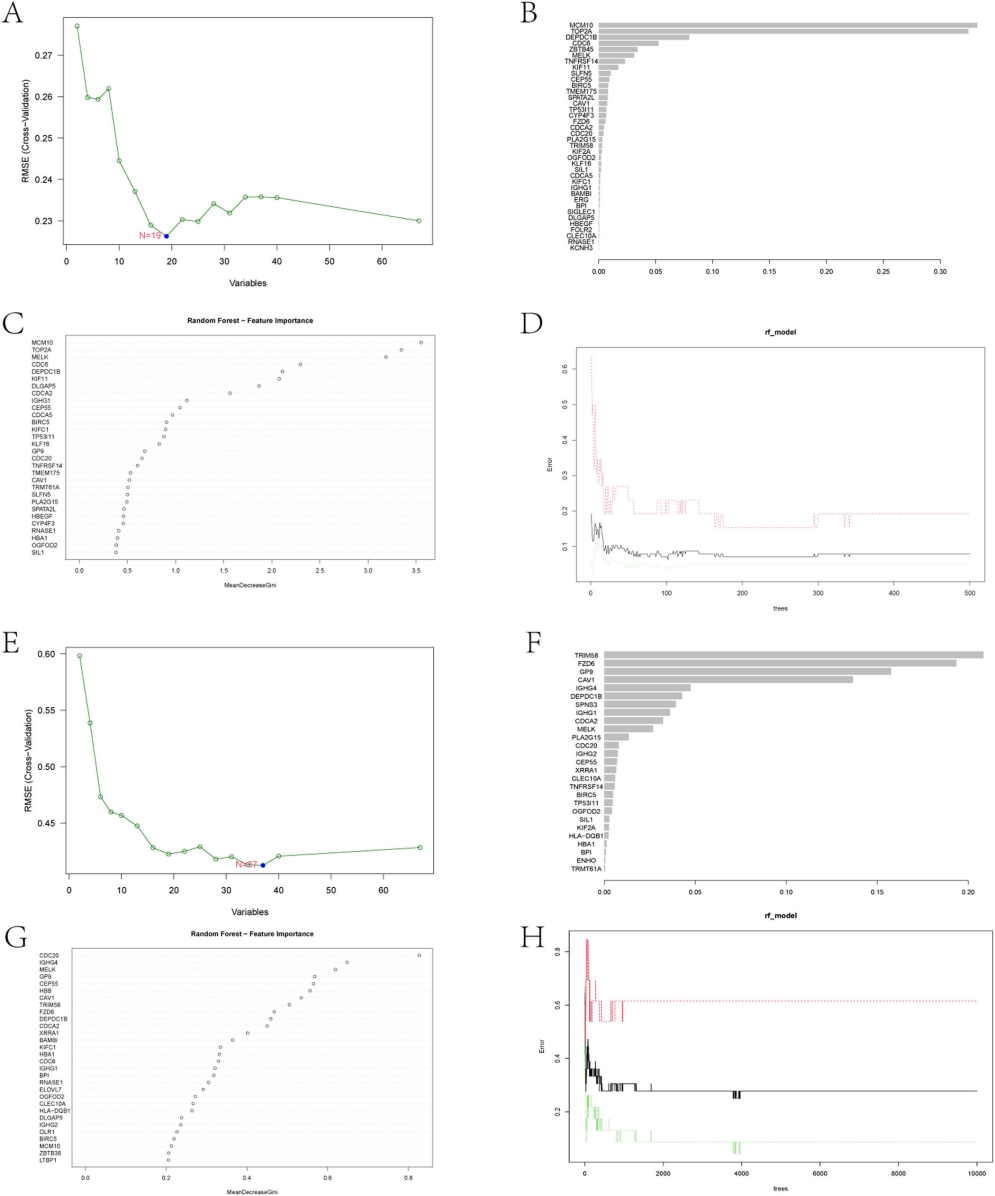
Machine learning algorithms for screening. SVM‐RFE algorithm for candidate genes between COVID‐19 (A) and long COVID (E). (D) Feature importance of candidate genes in XGBoost between COVID‐19 (B) and long COVID (F). (E) Ranking of the candidate genes via RF between COVID‐19 (C) and long COVID (G). (F) Relationship between the number of random forest and error rates between COVID‐19 (D) and long COVID (H).

**Figure 5:**
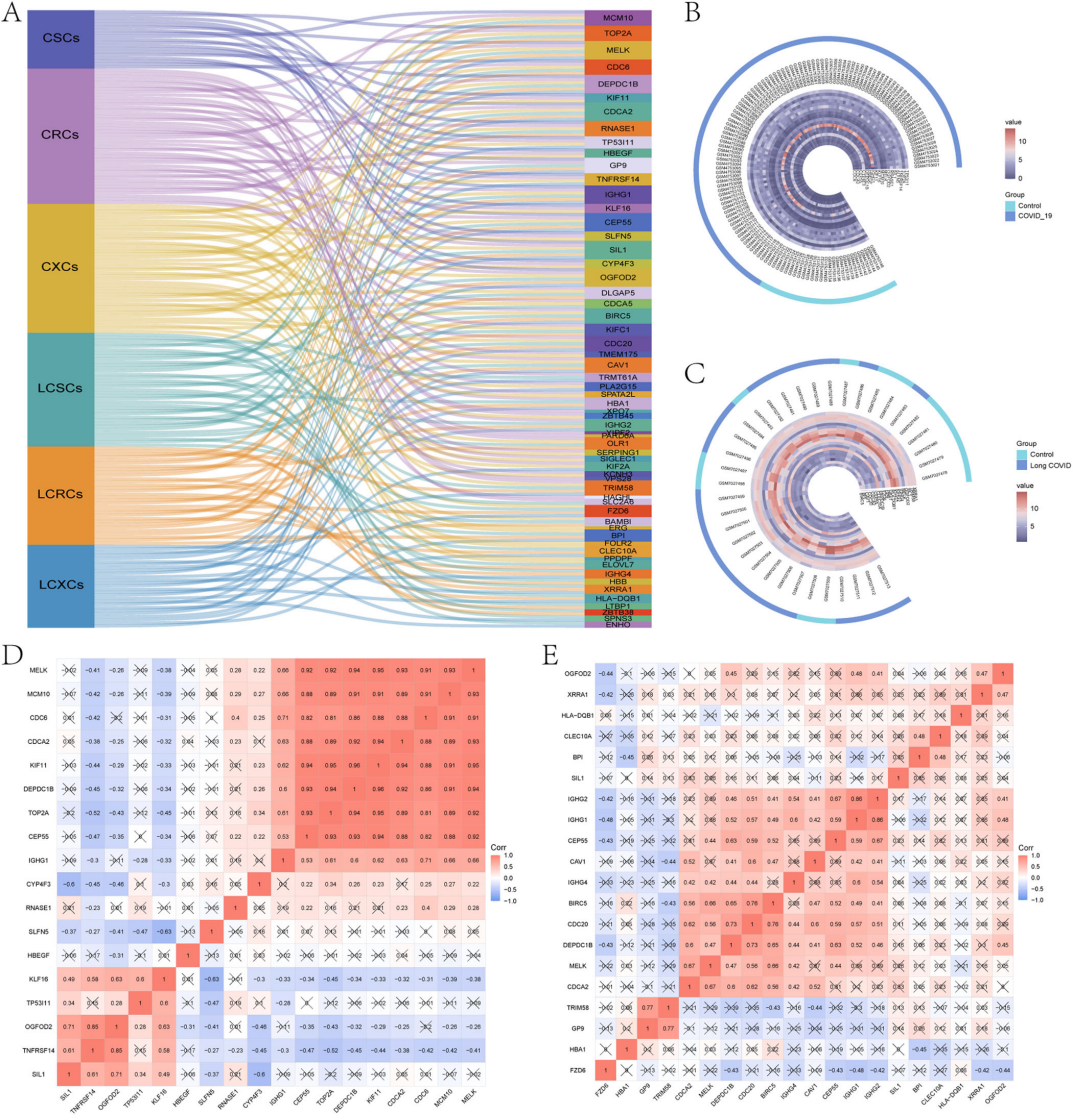
Correlation analysis and expression analysis of both COVID‐19 and long COVID result genes. Sankey diagram of result genes (A). Heatmaps of result genes in COVID‐19 (B) and in long COVID (C). Correlation analysis results from genes in COVID‐19 (D) and in long COVID (E).

XGboost, SVM‐RFE, and RF algorithms were performed for COVID‐19, which further investigated high performance for significant prediction in the development of COVID‐19 to long COVID of the candidate genes. Of those, 19 COVID‐19‐SVM‐RFE candidate genes (CSCs) were identified (Figure 4A), 44 COVID‐19‐XGBoost candidate genes (CXCs) (Figure 4B), and 42 COVID‐19‐RF candidate genes (CRCs) (Figure 4C,D). After analyzing the expression data of candidate genes in long COVID using XGboost, SVM‐RFE, and RF algorithms, we obtained 37 long COVID‐SVM‐RFE candidate genes (LCSCs) (Figure 4E), 27 long COVID‐XGBoost candidate genes LCXCs (Figure 4F), and 32 long COVID‐RF candidate genes (LCRCs) (Figure 5G,H), respectively. The intersection and visualization of the aforementioned results via a Sankey diagram yielded seven resultant genes: CEP55, CDCA2, MELK, DEPDC1B, IGHG1, OGFOD2, and SIL1 (Figure 5A; Supporting Information S15).

### 3.4 Identification and Validation of the Potential Biomarkers for Long COVID

**Figure 6:**
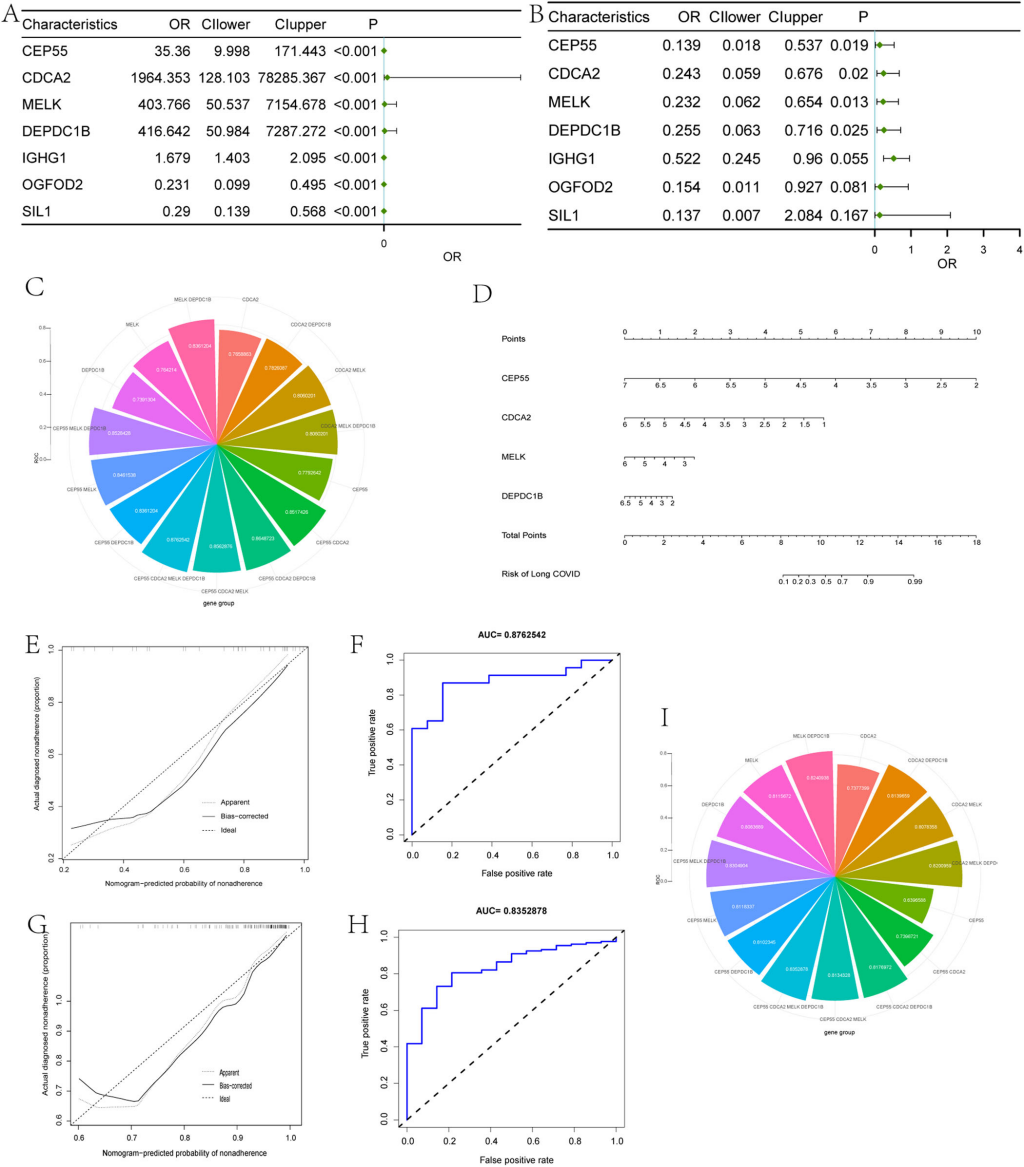
Construction and verification of a nomogram for long COVID risk. Logistic regression analysis of COVID‐19 (A) and long COVID (B). Polar area diagram of ROC for hub genes in GSE224615 (C) and GSE169687 (I). (D) Construction of a nomogram for predicting the risk of long COVID based on the hub genes. ROC curves of the combined hub genes model between GSE211378 (F) and GSE169687 (H). Construction of calibration curve for evaluating the predictive efficiency of the combined hub genes model between GSE224615 (E) and GSE169687 (G).

The resultant genes of candidate genes applied to the machine learning algorithms of COVID‐19 and long COVID were leveraged to assess the variability between disease samples and control samples (Figure 5B,C). Additionally, correlation analyses were conducted to investigate the interrelationships among the resultant genes (Figure 5D,E). Following the logistic regression analysis of the resultant genes, CEP55, CDCA2, MELK, and DEPDC1B were identified as hub genes (Figure 6A,B). ROC values of the hub genes for predicting the risk performance of long COVID were summarized (Figure 6C), and the joint prediction of CEP55, CDCA2, MELK, and DEPDC1B exhibited the best performance with an ROC value of 0.8762542 (Figure 6F). Eventually, a nomogram of risk predictors with the hub genes for long COVID was established (Figure 6D) and its predictive effectiveness was evaluated with a calibration curve (Figure 6E). We further verified the predictive performance of the hub genes in long COVID using the GSE169687 data set. It was found that predictive performance for the combination of CEP55, CDCA2, MELK, and DEPDC1B was also the highest (Figure 6I), with an ROC value of 0.8352878 (Figure 6H), and the related calibration curve was also remarkable (Figure 6G).

## 4 Discussion

The modular pharmacology platform, constructed based on module pharmacology, is utilized to investigate complex biological networks, considering which from the perspective of modules to measure and integrate networks [30]. At the back of the optimal modules selection based on Zsummary value, genes within these modules were interconnected in a network relationship and screened in combination with differential expression analysis. The results of enrichment analysis revealed that the pathogenesis of COVID‐19 and long COVID were primarily relevant to immunization; for instance, specifically in the immune cell infiltration analysis, the COVID‐19 sample group exhibited a significant number of differentially expressed immune cells. Several previous studies have reported that, for long COVID patients, there was a significant increase in the proportions of Tcm, Tfh, and Treg cells among CD4 + T cells [31], along with a relative rise in neutrophils and monocytes [32]. This shift promotes the conversion of proinflammatory M1 cells into M2 cells, suppressing proinflammatory cytokines and triggering an excessive anti‐inflammatory immune response [33]. Recent research projects [34, 35, 36, 37], including single‐cell transcriptomics, proteomics, and other analyses of the immune system in COVID‐19 recoveries, have indicated long‐term immune dysregulation following the transformation of COVID‐19 into long COVID. This observation aligns with the findings of our research.

Enrichment analysis suggests that the progression from COVID‐19 to long COVID is closely linked with immune‐related signaling pathways, providing potential evidence of disease transition mediated by the immune system. Machine learning algorithms dominate partially in life sciences, enhancing the reduction for time‐expenditure of data processing and the accuracy, reliability, and interpretability of data [38]. With the utilization of machine learning algorithms, the further investigation of candidate genes provided significantly optimized genes related to disease progression. After validation of independence, hub genes were approximately regarded as the most remarkable impact genes existing through the development of the study.

CEP55 regulates the processes of differentiation, proliferation, and migration of T cells and B cells in the immune system. It has been implicated in various studies reporting immune‐related diseases and tumors [39, 40]. The lack of SARS‐CoV‐2 virus genome replication and protein synthesis contributes to the stagnation of the host cell in an active metabolic state of the cell cycle, thereby facilitating the production of newly infected cells. However, CEP55 participates in mitotic exit and cytokinesis by recruiting PDCD6IP and TSG101 to the midbody during cytokinesis [41], regulated in expression upon the influence of viral replication, and synergistically contributes to the virus' proliferation. CEP55, by participating in cell division and regulating the cell cycle, influences tissue repair and renewal, particularly in high‐metabolic tissues such as immune and neural tissues. It may result in immune system fatigue, impair normal tissue repair, exacerbate fatigue, or affect pain perception through the modulation of inflammatory factor release. Wang et al. [42, 43] demonstrated CDCA2 protects against oxidative stress by promoting the expression of BRCA1 and NRF2, which inhibits the production of inflammatory factors and mitigates inflammatory reactions, delaying the onset and progression of inflammatory diseases. Pathophysiological processes, for instance, inflammatory immunity, dormant viral reactivation, endothelial damage, and coagulation abnormalities, which occur during the acute infection period of COVID‐19, lead to multi‐systemic sequelae in patients with long COVID [44, 45]. With inflammatory immunity dysregulated, latent viruses, for example, human herpesvirus 6, reactivate and drive neuroinflammation, provoking chronic fatigue syndrome (CFS) and contributing to symptoms such as fatigue, cognitive dysfunction, or sleep disorders in patients [46].

MELK, significantly associated with cell cycle regulation, proliferation, mitosis, and the assembly of splicing complexes, participates in regulating cell apoptosis and activating immune cells to produce a large number of cytotoxins and inflammatory factors, thereby eliminating infected or damaged cells [47, 48]. Dysfunction of MELK could account for prolonged activation of the immune system post‐infection, contributing to a chronic inflammatory state, which may activate or maintain the sensitivity of nociceptors, setting off chronic pain. Additionally, the increased demand for cellular repair attributable to prolonged infection and ineffective or energy‐depleting repair mechanisms may exacerbate fatigue in patients. Yang et al.'s study suggests that DEPDC1B activates the Wnt/β‐catenin signaling pathway, which reduces the adhesion of neutrophils and alveolar epithelial cells mediated by ICAM‐1/VCAM‐1, inhibiting the inflammatory response [49, 50]. DEPDC1B may hinder the repair or proliferation of neurons or glial cells, compromising nervous system health, exacerbating cognitive dysfunction, or causing abnormal transmission and amplification of pain signals, thereby triggering or intensifying pain perception.

The emergence of long COVID from the parallel or sequential actions of multiple systems in the organism makes it problematic to investigate its intricate biological mechanisms, dynamically assessing the functional status of affected systems, organs, and tissues related to symptoms, and precisely identifying clinical diagnostic and therapeutic targets for long COVID. In this study, we integrated the expression data of COVID‐19 and long COVID, identifying CEP55, CDCA2, MELK, and DEPDC1B through bioinformatics and modular pharmacological analysis. This discovery highlights the shared distinctive biomarkers and pathways between COVID‐19 and long COVID, providing potential targets for clinically diagnosing the transition from COVID‐19 to long COVID. At the same time, we conducted a preliminary exploration of the hub genes associated with the transformation of COVID‐19 into long COVID at the molecular level. We elucidated potential mechanisms of this transformation in terms of immunity and viruses, offering informative support for subsequent exploratory studies on the transition from COVID‐19 to long COVID.

This study has some limitations. Our analysis relies on data from the GEO database, which may introduce biases related to sample selection, data collection methods, and population variability. The gene expression data could also be affected by methodological discrepancies, potentially introducing batch effects and biases due to differing experimental conditions across data sets. Additionally, as this research is based on retrospective analysis, prospective cohort validation or in vivo and in vitro experimental studies are essential for further insights into the molecular mechanisms. The data sets utilized are not from patients who directly transitioned from COVID‐19 to long COVID but rather compiled data from multiple sources. Future comprehensive in‐depth studies on the immune mechanisms facilitating the transition from COVID‐19 to long COVID will employ proteomics, metabolomics, and single‐cell sequencing analysis. This research will provide fresh insights for clinical decision‐making and contribute to the development of novel therapies designed for the early prevention and treatment of long COVID.

## 5 Conclusion

This study, through bioinformatics and module pharmacology analysis, identified immune‐related subgroup biomarkers—CEP55, CDCA2, MELK, and DEPDC1B—associated with the progression from COVID‐19 to long COVID and preliminarily explained the immune‐related mechanisms of this transition. Our findings may offer potentially novel avenues for the early prevention, detection, and treatment of long COVID, informing future clinical decision‐making.

## Author Contributions

**Zhiyong Hou, Yu Ming:** data curation, formal analysis, software, writing–original draft. **Zhong Wang, Jun Liu:** methodology, writing–review and editing, funding acquisition. All authors read and approved the final manuscript.

## Ethics Statement

The authors have nothing to report.

## Conflicts of Interest

The authors declare no conflicts of interest.

## Supporting information

Supporting information.

Supporting information.

Supporting information.

Supporting information.

Supporting information.

Supporting information.

Supporting information.

Supporting information.

Supporting information.

Supporting information.

Supporting information.

Supporting information.

Supporting information.

Supporting information.

Supporting information.

## Data Availability

The data sets (accession number: GSE157103, GSE224615, and GSE169687) analyzed during the current study are available in GEO (https://www.ncbi.nlm.nih.gov/geo/), which are public functional genomics data repositories.
