# Supplementary material for: Potential Biomarkers for Predicting the Risk of Developing Into Long COVID After COVID‐19 Infection
Source: Immun Inflamm Dis. 2025 Jan 24;13(1):e70137. doi: 10.1002/iid3.70137 (PMC11760981; doi:10.1002/iid3.70137)
Supplement: Supplementary file 1 — Supporting information. [file IID3-13-e70137-s005.docx]

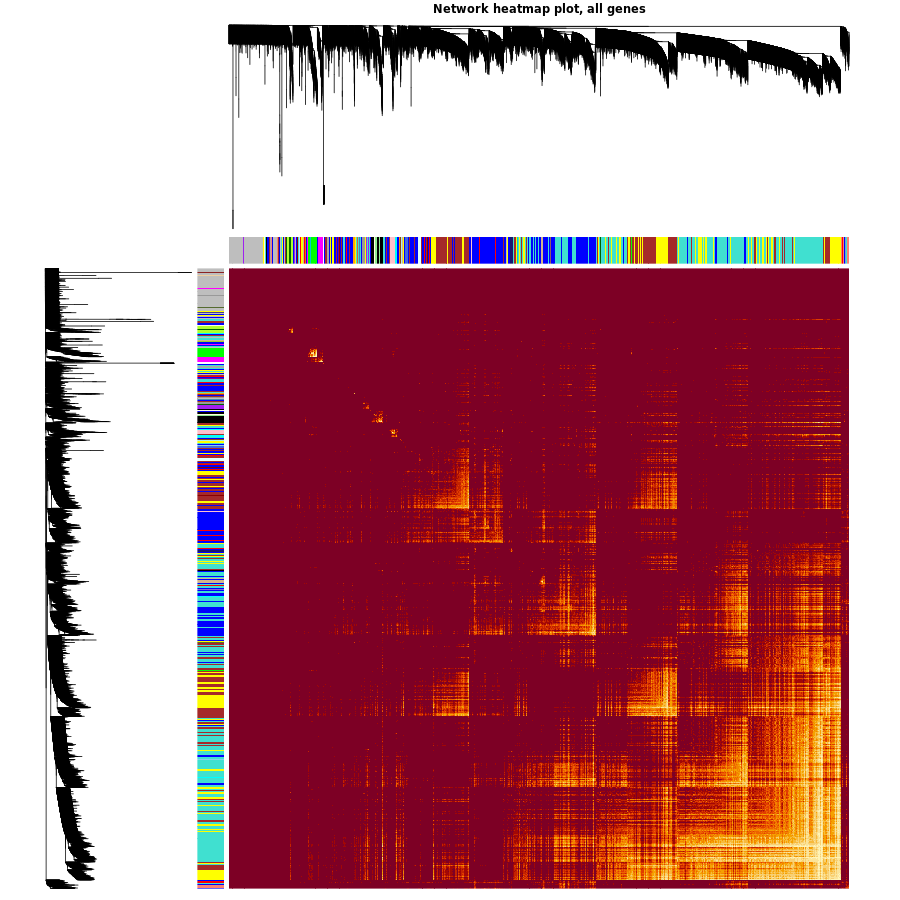


Heatmap of inter-module relationships for COVID-19


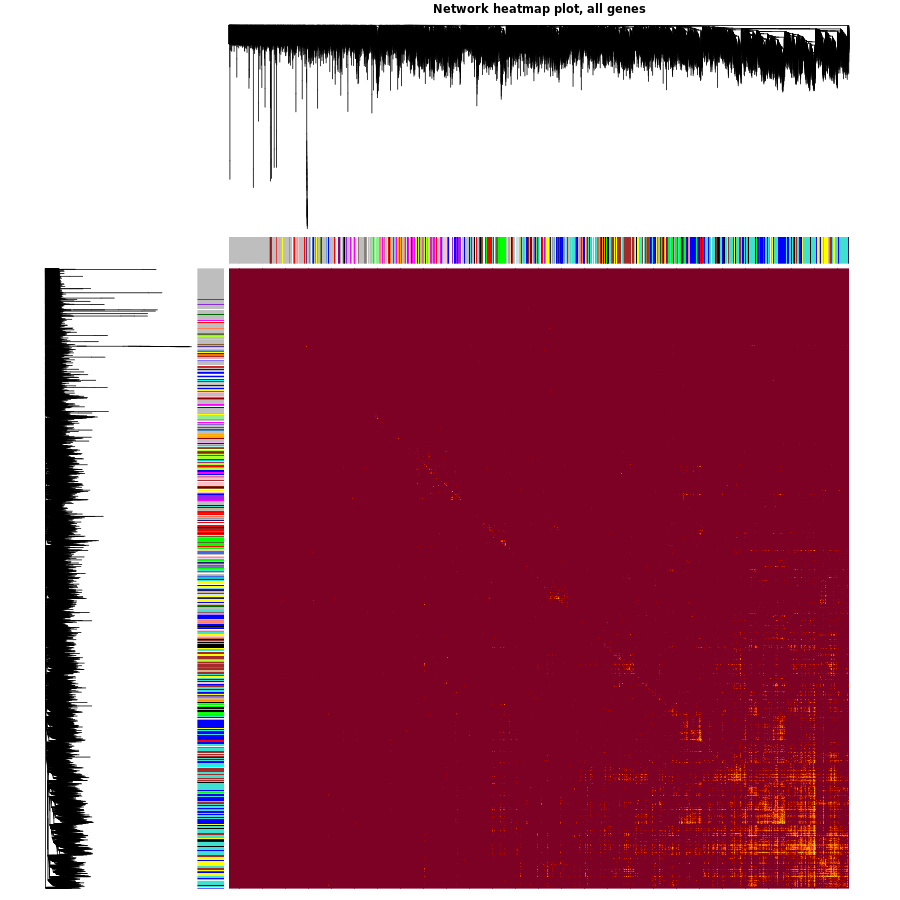


Heatmap of inter-module relationships for long COVID
